# Phase of care prevalence for prostate cancer in New South Wales, Australia: A population-based modelling study

**DOI:** 10.1371/journal.pone.0171013

**Published:** 2017-02-08

**Authors:** Xue Qin Yu, Qingwei Luo, David P. Smith, Mark S. Clements, Manish I. Patel, Dianne L. O’Connell

**Affiliations:** 1 Cancer Research Division, Cancer Council New South Wales, Sydney, Australia; 2 Sydney School of Public Health, the University of Sydney, Sydney, Australia; 3 Menzies Health Institute Queensland, Griffith University, Gold Coast, Queensland, Australia; 4 Department of Medical Epidemiology and Biostatistics, Karolinska Institutet, Stockholm, Sweden; 5 Nordic Information for Action eScience Center, Stockholm, Sweden; 6 Swedish e-Science Research Centre, KTH, Department of Mechanics, Stockholm, Sweden; 7 Discipline of Surgery, the University of Sydney and Department of Urology, Westmead Hospital, Sydney, Australia; 8 School of Medicine and Public Health, University of Newcastle, Newcastle, Australia; Texas Technical University Health Sciences Center, UNITED STATES

## Abstract

**Objective:**

To develop a method for estimating the future numbers of prostate cancer survivors requiring different levels of care.

**Design, setting and participants:**

Analysis of population-based cancer registry data for prostate cancer cases (aged 18–84 years) diagnosed in 1996–2007, and a linked dataset with hospital admission data for men with prostate cancer diagnosed during 2005–2007 in New South Wales (NSW), Australia.

**Methods:**

Cancer registry data (1996–2007) were used to project complete prostate cancer prevalence in NSW, Australia for 2008–2017, and treatment information from hospital records (2005–2007) was used to estimate the inpatient care needs during the first year after diagnosis. The projected complete prevalence was divided into care needs-based groups. We first divided the cohort into two groups based on patient’s age (<75 and 75–84 years). The younger cohort was further divided into initial care and monitoring phases. Cause of death data were used as a proxy for patients requiring last year of life prostate cancer care. Finally, episode data were used to estimate the future number of cases with metastatic progression.

**Results:**

Of the estimated total of 60,910 men with a previous diagnosis of prostate cancer in 2017, the largest groups will be older patients (52.0%) and younger men who require monitoring (42.5%). If current treatment patterns continue, in the first year post-diagnosis 41% (1380) of patients (<75 years) will have a radical prostatectomy, and 52.6% (1752) will be likely to have either active surveillance, external beam radiotherapy or androgen deprivation therapy. About 3% will require care for subsequent metastases, and 1288 men with prostate cancer are likely to die from the disease in 2017.

**Conclusions:**

This method extends the application of routinely collected population-based data, and can contribute much to the knowledge of the number of men with prostate cancer and their health care requirements. This could be of significant use in planning future cancer care services and facilities in Australia.

## Introduction

Prostate cancer is the most common non-skin cancer among Australian men, with 21,800 new diagnoses in 2009 [1], and as the population grows and ages it is expected that prostate cancer incidence will continue to increase [2]. Fortunately, earlier detection as a result of widespread prostate-specific antigen (PSA) testing and improved treatments mean that the majority of newly diagnosed prostate cancer patients will live for many years after their diagnosis [3], but with an expected increase in both incidence and years of life after diagnosis, the prevalence of prostate cancer will increase significantly in the future.

In a recent study, we showed that there will be 206,800 to 224,600 men living with prostate cancer in Australia by 2017 [4]. This prevalence estimate is a crucial but crude measure of the likely future health service requirements for cancer patients, as it includes people with a huge range of health service needs, from recently diagnosed patients requiring initial treatment to people who require extensive care and have severe disabilities from advanced disease, or long term cancer free survivors who need only minimal care. Therefore, in terms of providing a meaningful and useful measure to inform health care planning there is great benefit in segmenting the population of cancer survivors into different groups based on their health care needs [5]. Population-based studies identifying these different groups and planning for their health care needs are, however, currently relatively rare [6].

There are some inherent difficulties to modelling and predicting future health care requirements for prostate cancer patients, as the management of prostate cancer is a complex and sometimes controversial area of medicine. There are different options for managing and treating prostate cancer depending on the patient’s age, disease stage and general health status at diagnosis. Unlike other cancers, many men with prostate cancer may not receive aggressive treatment immediately after diagnosis because these radical treatments can have long-term adverse effects and more impact on patients’ quality of life than the cancer itself [7]. Recent patterns of care studies have shown that the main treatment options for prostate cancer include active surveillance (AS), surgery, radiotherapy (RT), androgen deprivation therapy (ADT) and watchful waiting (WW) [8,9,10,11,12]. AS and WW are management approaches unique to prostate cancer and are used to avoid or delay the need for radical treatments (surgery or radiotherapy), especially in situations where the risks of treatment are greater than the possible benefits. Reliable information on the proportions of patients receiving the various treatment options can be difficult to obtain, as much of this treatment can be delivered on an outpatient basis, limiting the availability of routinely collected data, and patterns of care studies tend to vary in how they group and define the less aggressive treatment options [8,9,10,11,12].

Age is an important factor in making treatment decisions for prostate cancer, with guidelines and studies suggesting that treatment decisions for elderly men are primarily based on age, as well as patients’ comorbidities and disease factors [10,13]. WW is considered to be an appropriate treatment option for older patients with less than a 10-year life expectancy [13], and while chronological age alone is not reliable in estimating life expectancy, studies do suggest that the patient’s age strongly influences treatment decision making [8] and is by far the strongest determinant for deferring treatment [11]. An Australian population-based cohort study (2013) found that while 80.8% of prostate cancer patients aged less than 75 years undertook active treatment within the first year of diagnosis, this proportion was much smaller for older men (58.3% of men ≥75 years had active treatment) [9], and substantial variation in treatment based on the patient’s age has also been reported by an American study which found that older men (aged > 75 years) with prostate cancer were more likely to be treated with ADT (40.8%) than those aged ≤ 75 years (9.3%), and were more likely to be under WW (18.9%) than their younger counterparts (4.7%) [8]. These treatment differences by age have relevance to the estimation of future health service requirements for prostate cancer patients, and thus age is an important factor to consider when modelling prostate cancer prevalence by phase of care.

Despite the complexities involved, the rising prevalence of prostate cancer means that developing methods for estimating future numbers of prostate cancer patients and their health service requirements is of increasing importance. By extending the application of routinely collected population-based data to develop a method for estimating the future number of prostate cancer patients requiring various levels of care we hope that this study may help to inform the planning of health service requirements in Australia. For this reason, we expanded our initial research on projecting prostate cancer prevalence in New South Wales (NSW), Australia [4] by dividing complete prevalence into phase of care prevalence with the use of additional data, including inpatient records, subsequent metastases, and causes of death.

## Materials and methods

### Ethical approval

This study involves analysis of routinely collected data and the records were de-identified (name, address, date of birth had been removed) before being provided to the research team. The NSW Population and Health Services Research Ethics Committee approved the use of the primary data from the NSW Cancer Registry (reference number: 2009/03/139) and the use of the linked cancer registry data with hospital records (reference number: 2010/04/223).

**Fig 1:**
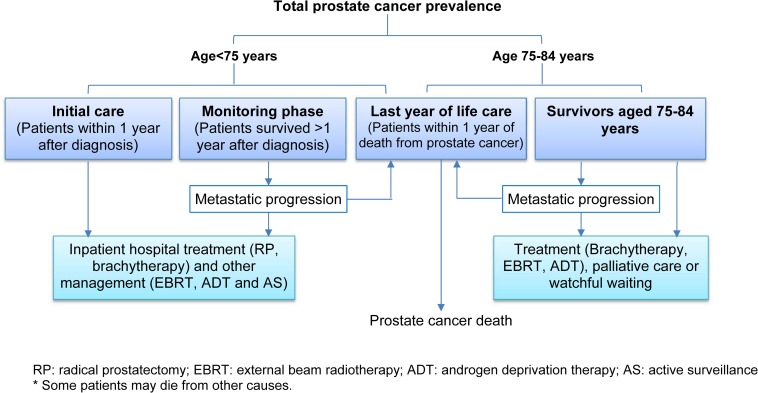
Prostate cancer phases of care. Patients who died from other causes were excluded from the prevalence count.

We have previously published a study of prostate cancer prevalence and future prevalence projections in NSW [4]. We used the population-based NSW Cancer Registry data and an illness-death model using the PIAMOD (Prevalence and Incidence Analysis MODel) software [4]. Prostate cancer data (ICD-O3 code—C61) [14] for men aged 18–84 years at diagnosis in 1996–2007 were used to project complete prevalence of prostate cancer in 2008–2017. We first modelled incidence (by fitting age-period-cohort models to obtain incidence projections) taking account of recent changes in PSA testing in NSW, and modelled survival (by fitting a mixture cure model to obtain survival projections), and then used the modelled incidence and survival estimates to calculate complete prevalence. These steps were illustrated in Fig 1 of the previous publication [4] and are included in Figure A in S1 Appendix. In the current study we further refined this prevalence modelling by grouping patients into more homogeneous groups that are likely to require similar levels of care.

To provide an estimate of inpatient care received during the first year of diagnosis, data for incident prostate cancer cases recorded in the registry in 2005–2007 linked with the NSW Admitted Patient Data Collection (APDC) for 2005–2008 were used to obtain inpatient data for prostate cancer specific procedures during the first 12 months after diagnosis. The APDC contains information on all inpatient separations (discharges, transfers and deaths), recorded as episodes of care, from all public, private and repatriation hospitals and private day facilities in NSW. Information collected for each episode of care includes both prostate cancer related and non-cancer related diagnosis codes and procedures codes [15]. In this study we only used data for prostate cancer related procedures to estimate the inpatient care needs for prostate cancer survivors.

### Prevalence by phase of care groups

In order to provide estimates of cancer care needs for prevalent prostate cancer patients, the previously estimated complete prevalence for the period 2008–2017 [4] was divided into more homogeneous groups according to age, years since diagnosis and cause of death (Fig 1). In the previous study we estimated two sets of projections of prostate cancer prevalence based on two different age-period-cohort incidence models (selected based on the model-fit-statistics combined with knowledge of the epidemiology of prostate cancer in Australia) to provide a reasonable range for the estimated future prevalence. For the sake of clarity, in this current study we used only one set of estimates generated from one age-period-cohort incidence model [4] and then divided this into care needs-based groups.

Due to the differences in treatment by age, and the lack of available data on the treatment options which are delivered on an outpatient basis, we looked at the phases of care in two age groups, and limited more detailed analysis of the prevalence of the specific inpatient treatment options to the younger age group only.

First, the complete prevalent cohort was divided into two groups based on patient’s age: men aged less than 75 years, and those aged 75–84 years for whom the benefits of radical treatment are more limited [16]. The care for those aged 75–84 years is a unique phase of care for prostate cancer (unlike cancers of the breast and colorectum [17,18]), with the majority of patients in this age group either receiving no active treatment or being treated with palliative intent [13], as indicated in Fig 1. We did not sub-divide this group further because the management patterns are generally similar throughout the entire post-diagnosis period.

The cohort of those aged less than 75 years was then further divided into two phase of care groups according to time since diagnosis, defined as the initial care phase (care provided in the first 12 months after diagnosis) and the monitoring phase (patients who have survived more than 12 months since diagnosis but are not in the last year of life prior to death from prostate cancer). Some men in the monitoring phase of the younger cohort would transition to the 75–84 years age group during follow-up when they turn 75 years old.

The final phase of care prevalence group was the ‘last year of life care’ phase, for which the annual number of deaths from prostate cancer was used as a proxy for the number of patients of any age requiring management of terminal prostate cancer. For each age group (<75 years and 75–84 years), the numbers of prostate cancer deaths per year for 2008–2017 were obtained by first calculating the average proportion of prostate cancer deaths over complete prevalence in 2005–2007, and applying this age-specific proportion to the corresponding projected complete prevalence for 2008–2017 (assuming stable survival trends). Detailed definitions of phase of care prevalence for other types of cancer can be found in previous publications [5,6,17,18,19,20].

Although over time each patient can contribute to more than one phase of care, at any one specific point in time a patient can only be in one phase of care.

### Estimating the proportion of patients receiving cancer-specific initial treatments

For patients aged less than 75 years we used the linked cancer registry and APDC data to estimate the proportions of curative treatments (radical prostatectomy (RP), brachytherapy) received as an inpatient in the first 12 months after diagnosis, as APDC data were found to be reasonably accurate for describing surgery and brachytherapy for men with prostate cancer [21]. To provide a stable estimate, we followed the 3 most recent cohorts (2005–2007) for at least one year after diagnosis to collect information on the care they received during the first year after diagnosis, and then applied the resulting proportions to the projected prevalence (<75 years) for 2008–2017, based on the assumption that the future patterns of care would not be substantially different from those of the most recent years for which data were available (2005–2008). The remaining cases were grouped together as ‘other treatment’, and mainly included AS, or external beam radiotherapy (EBRT) and ADT, which are primarily delivered to outpatients or via clinic visits [21].

### Estimating care requirements for patients with progressive disease

During follow-up after initial treatment some men may require more treatment due to tumour spread, so a sub-phase of care (treatment for metastases) was created to account for those cases. Cases diagnosed with a first primary prostate cancer in 1993–2007 were followed up for subsequent metastatic spread to the end of 2007. Episode data (consisting of notifications sent after initial diagnosis) from the registry were used for this analysis [17,18,22], and the development of subsequent metastases was identified using notifications from 120 days after the first diagnosis. The observed proportion (average of 2002–2006) with progression to metastatic disease in the cohorts of prostate cancer patients who survived more than a year in 2005–2007 were calculated for two prevalence groups: (1) patients in the monitoring phase, who were aged less than 75 years, and (2) elderly prevalence group, who were aged 75–84 years. When calculating rates of disease progression those who died from other causes were censored at the month of death. We then applied these observed proportions of metastatic cases to the corresponding projected prevalence groups in 2008–2017 to obtain the estimated number of future metastatic cases, assuming that the average risk in each age group is the same for the future as was observed in the historic data.

## Results

**Fig 2:**
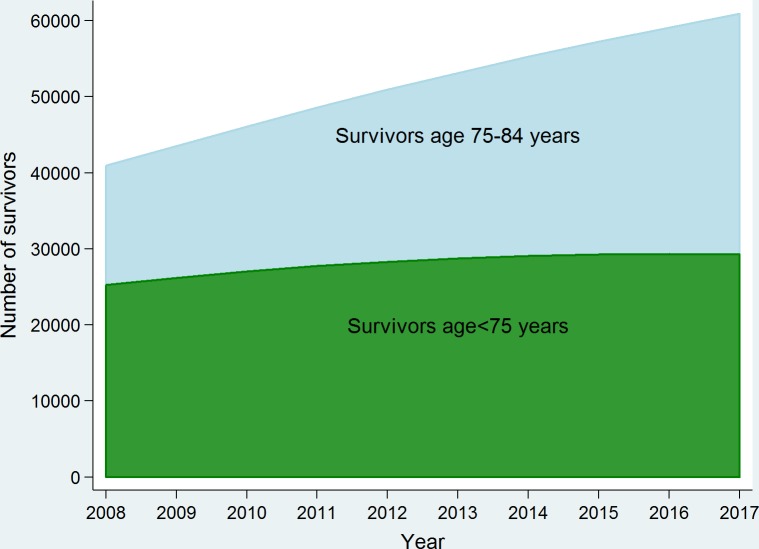
Trends in projected prevalence of prostate cancer in NSW, Australia 2008–2017.

The number of NSW resident men aged less than 85 years with a previous diagnosis of prostate cancer is projected to rise by about 60% in 10 years, from 38,322 in 2007 to 60,910 in 2017 [4]. The magnitude of this increase varies across age groups however, with a steeper increase among men aged 75–84 years, and a more moderate rise for those aged <75 years (Fig 2). As reported in the previous publication [4], the increased incidence rates should be the major contributors to this increase in prevalence along with population growth and ageing. Historical data indicated that the age-standardised incidence rates increased from 113.8 per 100,000 in 1996 to 157.6 per 100,000 in 2007, and our age-period-cohort model suggested that the increasing trend would peak at 181.8 per 100,000 in 2015, then start to drop slightly in 2016 (181.6 per 100,000) to 2017 (180.8 per 100,000). In contrast, observed mortality data showed that the annual number of deaths from prostate cancer decreased slightly over time, from 673 in 1996 to 622 in 2007 among men aged less than 85 years because of population growth and ageing.

Over time the proportion of men aged 75–84 years with prostate cancer increased, from 38.3% in 2008, to 50.4% in 2016 and 52.0% in 2017 (Fig 2), as a consequence of population ageing. Thus, this is projected to be the largest group of survivors (31,662) in 2017 (Table 1). For those aged <75 years, the vast majority of prostate cancer survivors will be in the monitoring phase (25,916) in 2017 (Table 1). Of those 25,916 men, 407 could be expected to progress to metastatic disease, while the corresponding number for the 75–84 years age group is 1,159. The proportions of men (aged < 75 years) being treated with RP and brachytherapy within one year after diagnosis were 41.4% and 6.0% in the observed data. The estimated number of men who would require these treatments for 2017 are also presented in Table 1. The corresponding estimated numbers for these treatments for 2008–2016 are included in the Table A in S1 Appendix.

**Table 1:** Estimates of numbers of patients within each phase of care in 2017, NSW Australia.

Phase of care category	Number of patients
Prostate cancer patients age 75–84 years*	**31,662**
Metastatic progression	1159
Prostate cancer patients age <75 years*	**29,248**
Total initial care	**3332**
RP	1380
Brachytherapy	200
Other treatments	1752
Total monitoring phase	**25,916**
Metastatic progression	407
Last year of life care†	**1288**
75–84 years	939
<75 years	349

* Alive at the end of 2017.

† died during 2017.

## Discussion

In this study we identified stratified pathways of care for men living with prostate cancer using data from a well-established, long-standing Australian population-based cancer registry, and estimated the likely magnitude of groups of survivors who may require prostate cancer services and care in the future. Such evidence-based information is essential for better understanding the number of men with prostate cancer and their likely demands on the health care system. This is a step forward in the use of routinely collected population-based data to provide policy and clinically relevant information about the future health service needs for patients at different stages of their cancer journey [5,17,18,19]. It is therefore a useful tool for planning future cancer care services and facilities, with the goal of improving the cancer experience for survivors, their caregivers and their families.

Our results indicate that men aged 75–84 years will constitute the largest group of prostate cancer survivors in 2017, with nearly 32,000 men in this phase in NSW alone, or equivalent to nearly 100,000 nationally. The available data on treatment options for this group of men are either not recorded or rarely captured in the APDC [21], but other studies suggest that ADT and WW are considered to be appropriate treatments [8,11,13], as radical treatment is not likely to improve these men’s long-term health outcomes [23]. Consistent with other studies [8], our linked data from the cancer registry and hospital records suggested that only 1.8% of those aged 75–84 years received RP during the first year after diagnosis (Table B in S1 Appendix). Our finding that in the future, older men with a previous diagnosis of prostate cancer will be such a significant proportion of the population of survivors, as well as the potentially large number of survivors aged more than 85 years [4] who are not included in this study highlights the importance of including the elderly population in studies of cancer survivors, and also suggests that the Australian health care system will need to be prepared to provide these older men with cancer care that meets their specific requirements.

Amongst men aged less than 75 years, our projections suggest that in 2017 there will be over 3000 men receiving initial treatment for prostate cancer in NSW. To further understand the inpatient hospital care requirements of this cohort of men we used linked data from the cancer registry and hospital records to quantify the number of men expected to receive specific treatments as an inpatient during the first year after diagnosis. It is however, difficult to compare our results to those from other Australian studies, as the most recent patterns of care studies did not report results stratified by age [9,11,12], and the classifications and groupings of treatment modalities can vary across studies (for example, one study grouped brachytherapy and EBRT together [11]). Nonetheless, our estimates of initial care, showing that approximately 41% of younger patients will receive RP, are broadly comparable with the most recent available Australian data, which showed that 42% of men received RP [11], although their estimates covered all age groups. Other main initial treatment options for this group of men, not captured by the inpatient data, may include AS and EBRT. A recent Australian study indicated that the patterns of care for men with prostate cancer have changed over time in two other Australian states (Victoria and South Australia), with the proportion of men being treated with RP staying relatively constant over time (38.6% in 2009 to 39.7% in 2013), while an increasing use of no active treatment (from 16.2% in 2009 to 21.6% in 2013) was matched with a decreasing trend in RT use (from 25.6% in 2009 to 15.6% in 2013) [11]. The balance between curative primary treatment and no active treatment, and the patient factors which influence these treatment decisions, has important implications for future health resource planning.

Our projections showed that in 2017 the monitoring phase will be the largest phase of care group amongst patients aged <75 years. As all the anticancer treatments for prostate cancer (RP, RT and ADT) are associated with a unique toxicity profile, men who have received these treatments require close monitoring and potentially extensive follow-up [16]. Unfortunately however, this phase of care is generally given little attention in clinical practice and research, where the focus is generally on initial diagnosis and curative treatment [24]. The healthcare needs of men in this phase may include, but are not limited to, prevention, detection and treatment of late effects, screening and treatment for recurrence and progression, and long-term counselling and support. The precise type of care required will depend on the initial treatment received, so that men who choose AS for their initial care will require continued monitoring to identify any sign of disease progression as early as possible, while for men who received treatment with curative intent as their initial care, close monitoring for side-effects of treatment and recurrence of the disease is required. For the latter group, the possible side–effects of radical treatment include urinary incontinence, sexual dysfunction, and bowel dysfunction for RP and RT [25], and ischemic heart disease, diabetes or osteoporosis for ADT [26], which can all be avoided or at least reduced with early intervention (for example, osteoporosis can be prevented with appropriate screening, lifestyle interventions, and therapy [27]). It is common to assume that once cancer survivors complete active treatment their health care needs are the same as the rest of the population [6], but there is evidence that in many countries some unmet needs continue to exist [28,29,30]. Studies from Australia and the UK showed that the areas of greatest unmet supportive care need for men with prostate cancer were for psychological distress, sexuality-related issues and management of enduring lower urinary tract symptoms [28,29]. A recent European study found that over 80% of men surveyed reported some type of unmet supportive care needs including psychological, sexual and health system and information needs [31]. It is also noted that interventions to improve coping strategies for this group of men may be as important as medical interventions [32]. As the healthcare needs of men in this phase of care are so varied, and that the number of men in this phase of care is likely to continue to grow, planning for the impact this will have on the healthcare system is highly complicated and yet also extremely important.

The inclusion of data on subsequent metastatic disease progression is crucial to the accurate estimation of phase of care prevalence, as this information is not routinely collected in population-based cancer registries [19,29] and most studies have to rely on indirect methods to estimate the population with metastatic disease. One UK study used mortality data to estimate the number of patients with metastatic prostate cancer, but this number included both patients initially diagnosed with metastatic disease and those with subsequent diagnoses [19]. Our estimated proportion (2.6%) with subsequent metastases was broadly comparable with that reported for an American cohort (2.8%) [33], although the patients’ characteristics and length of follow-up were somewhat different from ours. Most of the older men with subsequent metastatic disease would need ADT, palliative radiotherapy or bisphosphonate therapy as well as chemotherapy, while aggressive treatment may be used for patients with life expectancy of more than 10 years.

Men who die from prostate cancer will require care in the last year of life, which generally involves improving quality of life through appropriate oncological treatment and the control of symptoms such as bone pain, weight loss, fatigue and anxiety and depression [34]. Other symptoms may include urinary outflow obstruction, weakness due to spinal cord compression, lymphoedema and anaemia. Bone pain is the most common symptom among patients with metastatic prostate cancer and the main treatment is oral analgesia [35]. Appropriate anticancer treatment (surgery, radiotherapy, ADT) can be very useful in the management of pain and other symptoms [34]. This is an important period for health service planning, as patients’ general health tends to deteriorate rapidly in the last year of life, so that their health care needs are likely to increase substantially not only for cancer-specific care but also for other health issues.

This study has some limitations. First, the APDC data captured inpatient care only, so that data on ADT or chemotherapy and visits to general practitioners and other outpatient clinics are not captured, and data on EBRT are very incomplete. In addition, these estimates of initial care needs are based on past data (up to 2008), so if the patterns of care for prostate cancer have changed over time they will not accurately reflect current or future care needs. Nonetheless, our estimates of the proportion of men treated with RP (41%) were broadly comparable with the most recent Australian data (42%) [11]. While acknowledging the limitations of the records linkage with the APDC, the use of APDC data does for the first time enable us to provide valuable information on some of the potential future care requirements of the prevalent population. Second, as we have previously acknowledged [4], there is considerable uncertainty in the projected complete prevalence as it is based on assumptions. This, combined with the uncertainty regarding the choice of treatment options, makes the prediction of future demands for care variable and potentially unreliable. However, this does not mean that these projections are not plausible propositions based on the observed data [36], and it is hoped they may aid the planning of prostate cancer care requirements and further research in this field. Thirdly, the definition of our monitoring phase is very broad, including a wide range of care needs from those who just completed radical treatment (who may need recovery and readjustment after the treatment), to those still under conservative treatment (AS/WW), or those who have survived up to five years since initial diagnosis to those long-term survivors who may need minimal prostate cancer care. It would be desirable to be able to divide the monitoring phase into more specific phases to accommodate these different care needs-based groups accurately if more detailed treatment and follow-up data were available. Finally, the continuum from initial diagnosis and treatment to post treatment monitoring and last year of life care is oversimplified. Although the phase of care categorization used here is useful, the processes are not so discrete, and some of them intersect or transition between states, as there are many different possible paths cancer patients may experience from diagnosis to survival or end of life.

This method extends the application of routinely collected population-based data, and can contribute much to the knowledge of the number of men with prostate cancer and their health care requirements. The results from this study also suggest that there is a need to take action now to collect more detailed data on disease stage, tumour characteristics, PSA levels and care received as well as severe late effects of cancer treatment, to quantify the number of prostate cancer patients at different stages of their cancer journey. We hope this study will stimulate further research into refining the care phases using additional data, so that the future health care requirements of prostate cancer patients can be fulfilled.

## Supporting information

S1 Appendix (XLSX)
